# Possible role of hyperimmunoglobulin in reducing the risk of maternal–fetal transmission of cytomegalovirus in the valacyclovir era: a case series

**DOI:** 10.1007/s00404-026-08432-0

**Published:** 2026-04-27

**Authors:** Anna Barbiero, Daniele Lilleri, Piera d’Angelo, Federica Zavaglio, Matilde Tavanti, Sara Biagioni, Alessandra Ipponi, Michele Cecchi, Beatrice Borchi, Michele Salvatore Trotta, Lucia Pasquini, Alessandro Bartoloni, Lorenzo Zammarchi

**Affiliations:** 1 Department of Experimental and Clinical Medicine, University of Florence, Florence, Italy; 2 Microbiology and Virology, Fondazione IRCCS Policlinico San Matteo, Pavia, Italy; 3 Hospital Pharmacy and Pharmaceutical Policies, Careggi University Hospital, Florence, Italy; 4 Infectious and Tropical Diseases Unit, Careggi University Hospital, Florence, Italy; 5 Fetal Medicine Unit, Careggi University Hospital, Florence, Italy

**Keywords:** Pregnancy, CMV, Congenital CMV, Immunocompromised, Valacyclovir, Hyperimmunoglobulin

## Abstract

**Introduction:**

Valacyclovir is the only treatment option during pregnancy which has been demonstrated to be effective within a randomized clinical trial for prevention of transplacental cytomegalovirus (CMV) transmission. However, the use of high dose intravenous hyperimmunoglobulin (HIG) could reduce the rate of vertical transmission according to some observational studies.

**Cases presentation:**

We report three peculiar cases in which high dose HIG was administered in substitution to or in addition to valacyclovir to reduce the risk of transplacental transmission of CMV. Two were immunocompromised pregnant women—one with recurrent CMV reactivations due to solid organ transplant-related immunosuppression and one with primary CMV infection and lack of IgG production due to anti-CD20 treatment for multiple sclerosis—in which HIG was co-administered with valacyclovir. The third case involved an immunocompetent pregnant woman to whom HIG was administered in substitution to valacyclovir due to severe gastrointestinal side effects related to the latter medication. In all cases, the treatment was well tolerated and the newborns tested negative for CMV at birth.

**Conclusion:**

Together, these cases give an interesting perspective on the possible role of HIG in selected immunocompromised pregnant women with primary and non-primary CMV infection in addition to valacyclovir, and in immunocompetent pregnant women unable to tolerate valacyclovir or in whom the drug is contraindicated.

## What does this study add to the clinical work

**Table:** 

CMV-specific hyperimmunoglobulin could find application in substitution to or association with valacyclovir for treatment of CMV infection during pregnancy in specific situations, such as severe immune suppression or valacyclovir intolerance or contraindication	

## Introduction

Congenital Cytomegalovirus (CMV) is the most common perinatal infection worldwide, affecting approximately 0.5%-2.5% of newborns worldwide, and representing the leading cause of non-genetic sensorineural hearing loss and potentially severe and permanent neurological disability [1, 2].

CMV infection during pregnancy can be primary or non-primary, the latter resulting from viral reactivation or reinfection with a distinct strain [3]. Primary infection is associated with a transplacental transmission rate of 30–40%, resulting in 10–15% of symptomatic newborns at birth. Among them, about 50% will be affected by long term sequelae. Of the remaining 85% of asymptomatic newborns, 10–15% will also develop long term sequelae within the first 5 years of age [4]. The risk of neurological sequelae appears to be predominantly associated with infections acquired in the peri-conceptional period or during the first trimester of pregnancy [5]. Non-primary infections have a lower risk of transplacental transmission (0.2%-1.5%), although the risk of fetal-neonatal sequelae is comparable once transmission has occurred [6].

In Western Europe, 52% of congenital CMV cases are due to primary maternal infection, while 48% result from non-primary infection [1].

Until recently, no effective therapies were available to reduce the risk of intrauterine CMV transmission in case of primary CMV infection during pregnancy. However, significant innovations have emerged in recent years.

A randomized clinical trial showed that high-dose valacyclovir (VCV) administered after primary maternal CMV infection acquired in early pregnancy reduces vertical transmission by 70% [7]. These data have been replicated in some non-randomized observational studies [8–10].

High dose CMV hyperimmunoglobulin (HIG) has also been investigated to mitigate the risk of maternal–fetal transmission, although conflicting results, higher costs and the need for repeated intravenous infusions have limited its use in clinical practice [11–13]. Nevertheless, HIG may represent a therapeutic option in selected clinical scenarios. We report three cases of CMV infection in pregnancy in which the use of HIG, indicated due to different clinical reasons, could have contributed to reduce the risk of vertical CMV transmission.

## Methodology and case selection

We included in this case series all pregnant patients evaluated at the Tuscany Regional Referral Center for Infectious Diseases in Pregnancy (TRRCIDP), Careggi University Hospital (Florence, Italy), for whom authorization for off-label use of HIG was requested to the hospital pharmacy for the reduction of the risk of congenital CMV. Cases were included starting from 2020, when VCV was formally added to the list of National Health Service reimbursable drugs for the prevention and treatment of congenital CMV during pregnancy.

Selection criteria for HIG administration were:

- Primary CMV infection for which the use of valacyclovir is contraindicated or not tolerated due to adverse reactions.
- Primary or non-primary CMV infection in immunosuppressed pregnant women in whom the endogenous immunological response is documented to be inefficient.

Due to their low frequency and specific clinical characteristics, no diagnostic or virological monitoring algorithms are available in our center, where a personalized approach is usually adopted for such cases.

### Microbiological assays

CMV-specific IgG and IgM antibody detection was performed with the Alinity i CMV IgG and IgM assays (Abbott Laboratories, Abbott Park, IL, USA), or using LIAISON XL (Diasorin, Saluggia, Italy) according to the manufacturer’s instructions, whereas CMV IgG avidity was assessed using the CHORUS Cytomegalovirus IgG Avidity assay (Diesse Diagnostica Senese, Monteriggioni, Italy), following the manufacturer’s protocol.

CMV-specific antibody response was assessed through a neutralization assay and antibody-dependent cellular cytotoxicity (ADCC). For the neutralization assay, we used VR1814 for epithelial cells (ARPE-19, ATCC) and AD169 (ATCC, Manassas, VA, USA), a reference laboratory-adapted strain, for fibroblast cells (HELF, isolated in-house). Virus (at a concentration of 100 focus-forming units per well) and human serum antibody mixtures were added onto monolayers of both types of cells in 96-well plates. After incubation for 48 h at 37◦C and 5% CO2, cells were fixed and stained using a p72-specific murine mAb (produced in-house). The serum dilution inhibiting virus infectivity by 50% was considered the 50% neutralizing antibody titer.

The percentages of CMV-specific T cells were measured following incubation of peripheral blood mononuclear cells (PBMC) with autologous, monocyte-derived, HCMV-infected (strain VR1814, a clinical isolate) dendritic cells (DC) by intracellular determination of IFNγ production. PBMCs were stained with the following mAbs (all from BD Biosciences, San Jose, CA): CD8 mAb V500, CD3 PerCP-Cy5.5, CD4 APC-Cy7, IFN-γ PE-Cy7, IL-2 APC, CD45RA FITC, and analyzed with a FACS Lyric flow cytometer and FACSuite software.

Absolute CD3 + CD4 + and CD3 + CD8 + counts were measured in whole blood samples by flow cytometry (BD Multitest CD3/CD8/CD45/CD4 with BD TruCOUNT Tubes, BD Biosciences). The total number of HCMV-specific CD4 + and CD8 + T-cells was calculated by multiplying the percentage of HCMV-specific T-cells positive for IFN-γ by the relevant absolute number of CD4 + and CD8 + T-cell counts.

Cell-surface expression of CD107a was used as a marker for NK cell degranulation and therefore activation. ARPE-19 cells, infected for 120 h with VR1814 at a MOI of 10, were co-cultured for 4 h with cytokine-activated PBMC (from a single CMV-seropositive donor) in the presence of serum and CD107a BV510 (BD Biosciences). PBMC were then stained with CD56 PeCy7, CD3 PerCP-Cy5.5, CD57 Pacific Blue (Beckman Coulter, Brea, California, US), and NKG2C APC (Bio-techne, Minneapolis, MN, US). Using a FACS Lyric flow cytometer and FACSuite software, the percentage of CD107a-positive CD3-CD56 + or CD3-CD56 + CD57 + NKG2C + cells was determined, as already described [14, 15].

CMV polymerase chain reaction (PCR) was performed using the CMV Elite MGB Kit (ElitechGroup, Puteaux, France) following the manufacturer’s protocol.

## Cases description

The main features of the reported cases are summarized in Table 1.

**Table 1:** Main clinical features of the reported cases

	CMV infection type/timing	Comorbidities	Condition determining HIG indication	Treatment regimen	Virological monitoring after treatment initiation	Foetal investigations	Neonatal outcome
Case 1	Primary CMV infection acquired 2 years before conception. Persistent replication during the periconceptional period and in early pregnancy	Previous liver transplant on treatment with tacrolimus and azathioprine	Fluctuant CMV-DNA positivity in blood or urine in the peri-conceptional period; reduction in CMV-specific CD4 + T-cells response	VCV 2g qid combined with HIG 200 IU/kg biweekly from beginning of pregnancy, until delivery	Low level CMV-DNA intermittently positive in saliva or urine during first trimester (negative in blood), persistently negative in blood, urine and saliva from second trimester until delivery	- Amniocentesis at week 20: declined - Second-level fetal ultrasound monitoring until delivery: normal	Negative CMV-DNA in neonatal blood, saliva and urine at birth
Case 2	Primary CMV infection, first trimester	None	Severe VCV gastrointestinal intolerance	VCV 2g qid from week 11 to 13, followed by HIG 200 IU/kg on weeks 16, 18 and 20	CMV-DNA negative in blood, urine and saliva at week 20	- CMV-DNA on amniotic fluid at 20^+3^ weeks: negative - Second-level fetal ultrasound monitoring until delivery: normal	Negative CMV DNA in neonatal blood, saliva and urine at birth
Case 3	Primary CMV infection, likely during first trimester	Multiple sclerosis on treatment with ocrelizumab	Marked reduction in CMV-specific CD19 + B lymphocytes and negative CMV-specific IgG	VCV 2g qid from week 23 until delivery, combined with HIG 200 IU/kg on weeks 25 and 27	CMV-DNA positive in saliva or urine at diagnosis, negative between 24–29 weeks, and re-detected in urine thereafter; persistently negative in blood	- Second-level fetal ultrasound until delivery: normal - Amniocentesis not recommended	Negative CMV DNA in neonatal blood, saliva and urine at birth

### Case 1

In January 2022, a 32-year-old woman with a history of ulcerative colitis and sclerosing cholangitis requiring hepatic transplant in 2017, was evaluated at the TRRCIDP, Careggi University Hospital (Florence, Italy) before undergoing a medically assisted procreation procedure due to recurrent CMV reactivations.

In 2020, she was diagnosed with primary CMV infection causing prolonged fever, hepatitis and pancytopenia and she was treated with a 3-week course of ganciclovir followed by valganciclovir (VGC) secondary prophylaxis.

Upon evaluation at the TRRCIDP the patient was still on VGC prophylaxis and maintenance immunosuppressive treatment with tacrolimus and azathioprine. CMV serology showed positive IgG with high avidity (63%, considered high if > 40%) and negative IgM. Flow cytometry showed absent CMV-specific CD4 + T-cell response (0.0 cells/µL) but detectable CD8 + response (2.36 cells/µL). CMV-DNA was not detected in blood. VGC and azathioprine were discontinued due to potential teratogenicity for the former and to reduce the degree of immunosuppression for the latter, and prophylaxis with CMV-specific hyperimmunoglobulin (HIG) 100 IU/kg biweekly (standard dosage, approved for solid organ transplant recipients) was initiated in April 2022.

However, in June 2022, CMV-DNA was detected in blood and saliva. Therefore, VCV (2g qid) was added to reduce the risk of CMV vertical transmission before the initiation of the medically assisted procedure, but viral replication in blood, urine and saliva persisted intermittently in the following months.

In December 2022, HIG and VCV treatment were discontinued with the aim of allowing CMV replication and theoretically stimulating the immune response against CMV. Further waiting for the medically assisted procedure was recommended.

By May 2023, both CMV-specific CD4 + (1.03 cells/µL) and CD8 + (31.05 cells/µL) T-cells were detected on flowcytometry, but fluctuant positivity of CMV polymerase chain reaction (PCR) on blood or urine was observed in the following months.

In March 2024, she reported a positive pregnancy test following natural conception. In the meantime, immunophenotyping showed a drop in CMV-specific CD4 + T-cells (0.35 cells/µL), so off-label high dose CMV-specific HIG 200 IU/kg biweekly was started, in combination with VCV (2g qid) for the duration of the pregnancy. The patient declined amniocentesis at 20 weeks.

During April and May 2024, CMV PCR results were intermittently positive in saliva and urine, with viral copy numbers below the detection limit, while remaining persistently negative in blood. From July 2024 onward, CMV PCR on all biological samples became consistently negative. Obstetric and fetal ultrasound follow-up remained normal throughout pregnancy.

She gave birth on November 17th, 2024. The newborn tested negative for CMV by PCR on urine, saliva, and blood during the first week of life, and his neurodevelopmental status was normal at 7 months of age.

### Case 2

The second case involves a 31-year-old woman evaluated at the TRRCIDP in April 2024 due to a primary CMV infection diagnosed at the 10th week of gestation. Tests performed at the TRRCIDP confirmed positive anti-CMV IgG and IgM, with low IgG avidity, and positive CMV PCR on peripheral blood. VCV (2g qid) was initiated at the 11th week of pregnancy, and second-level ultrasounds were recommended. However, due to severe gastrointestinal intolerance, she stopped the treatment about two weeks after initiation. Subsequently, off-label treatment with CMV-specific HIG at a dosage of 200 IU/kg every two weeks was administered (at the 16th, 18th and 20th weeks of pregnancy) until amniocentesis, performed at a gestational age of 20 weeks and 3 days. Amniotic fluid was negative for CMV-DNA; at the same time, CMV PCR on peripheral blood, urine and saliva was negative. The second-level ultrasound monitoring was normal in all the follow-up controls. The newborn was in good health and had a negative CMV PCR on saliva, blood and urine at birth.

### Case 3

The third case involves a 35-year-old pregnant woman with multiple sclerosis treated with the anti-CD20 monoclonal antibody (ocrelizumab) every eight months. The treatment was discontinued six months before her last menstrual period, when the patient had expressed her desire for pregnancy.

In January 2025 (gestational age of 12 weeks and 5 days), she was referred to the TRRCIDP for suspected primary CMV infection (CMV IgG negative, CMV IgM borderline). Repeated blood tests revealed negative CMV IgG and IgM, and CMV-DNA below the lower limit of quantification (< 254 copies/mL). These results were interpreted as a false positive finding, and she was recommended to repeat the tests monthly until the 20th week of pregnancy.

On March 28th, 2025, the patient was again referred to the TRRCIDP due to isolated IgM positivity (confirmed by two different assays) and negative CMV IgG. CMV-DNA was negative in peripheral blood but positive in saliva (< 500 copies/mL) and in urine (2059 copies/mL). In light of these results, antiviral therapy with VCV 2g qid was started on April 9th, 2025 (gestational age of 23 weeks). Given the gestational age and the absence of sonographic signs of fetal distress or infection, invasive investigations were not recommended.

In mid-April, CMV IgG was persistently negative; moreover, hypogammaglobulinemia was noted, showing reduced levels of IgG (5.64 g/L; normal range: 7.00–16.00 g/L) and IgA (0.51 g/L; normal range: 0.70–4.00 g/L), while IgM levels remained within normal limits. Specific lymphocyte immunophenotyping on peripheral blood showed a marked reduction in CD19 + B lymphocytes (9 cells/µL; normal range: 163–288 cells/µL), normal CD4 + and CD8 + counts, and the presence of a CMV-specific T-cell response (CD4 + 5.42 cells/µl and CD8 + 0.59 cells/µl, both considered positive if ≥ 0.4 cells/µl) with values consistent with primary CMV infection (i.e., low frequency of IL-2 producing CD4 + and CD8 + T cells) [14].

**Fig. 1:**
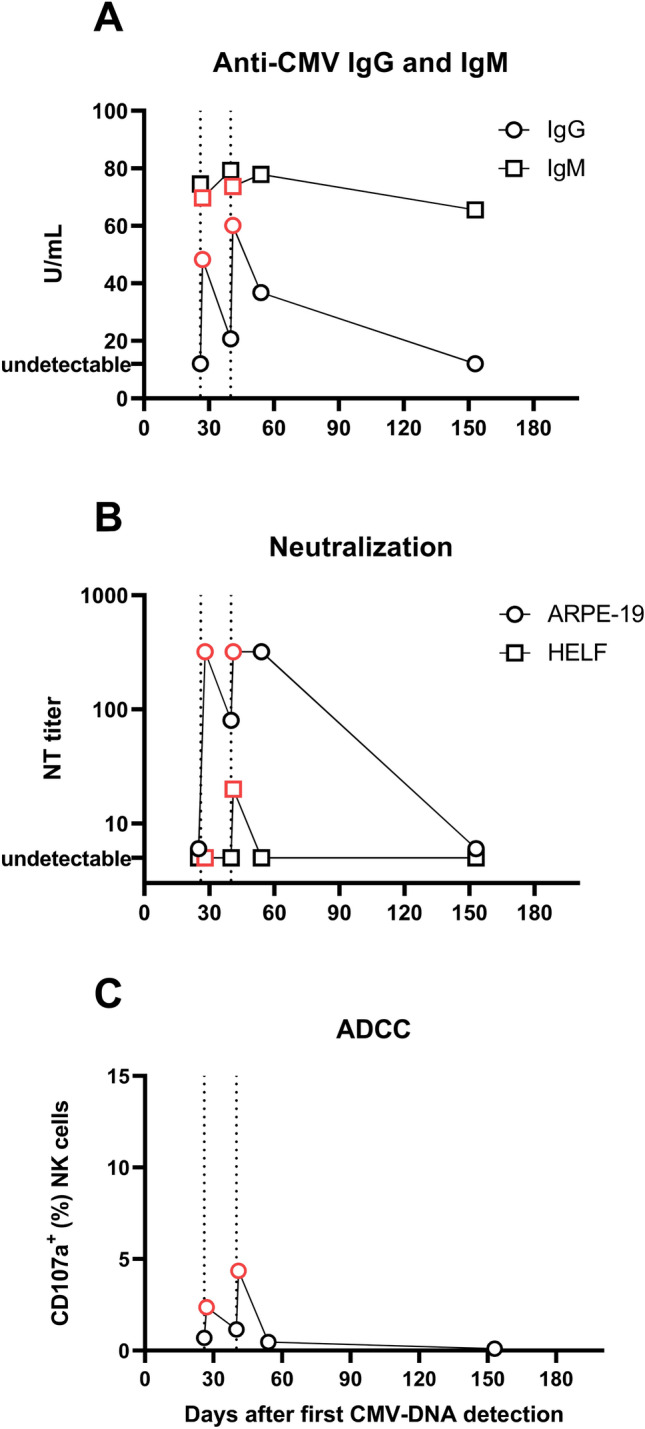
CMV-specific antibody levels and activity in case 3. Anti-CMV IgG (circles) and IgM (squares) levels at different days after the first detection of CMV-DNA are shown in panel A. The serum neutralization titer against the infection of epithelial (ARPE-19, circles) and fibroblast (MRC-5, squares) cells and the antibody-dependent cell-cytotoxicity (ADCC) against the infection of ARPE-19 cells are shown in panels B and C, respectively. Vertical dotted lines represent the days of the HIG administration. Red points illustrate tests performed right after HIG administration

Due to the impaired humoral immunity and the very low count of CD19 + B lymphocytes, high-dose CMV-specific HIG (200 IU/kg) was administered at 25 and 27 weeks of gestation. Following HIG administration, transient CMV IgG positivity (with high avidity) was observed (Fig. 1), although followed by a decline two weeks later. IgG levels rose again immediately after the second HIG administration and dropped two weeks later. Serum neutralizing activity against epithelial cell infection was observed after HIG administration, while a weak neutralizing activity against infection of fibroblast cells was observed only after the second HIG administration. Moreover, weak but detectable ADCC [15] was also detected after HIG administration. CMV-DNA in urine, saliva, and blood remained negative until May 20th, 2025 (gestational age: 29 weeks). However, in subsequent controls, CMV IgG again decreased to negative levels (with concomitant disappearance of ADCC and neutralizing activity) and CMV-DNA was detected in urine (1466 copies/mL). Antiviral therapy was maintained until the end of pregnancy. Second-level ultrasound screenings and fetal MRI showed no significant abnormalities. She gave birth at term, and the newborn was asymptomatic and tested negative on urine and saliva at birth.

## Discussion

VCV is currently the only recommended therapeutic option in case of primary CMV infection in early pregnancy. At high doses (8 g/day), it has shown a reduction in the risk of vertical transmission of up to a 70% when used in pregnant women with primary CMV infection during the first trimester, and its use is currently recommended by several national and international guidelines [7, 16]. Despite a favorable tolerance profile, its increasingly widespread use has inevitably uncovered a small proportion of subjects in whom the medication is not tolerated, as described in Case 2. In these cases, no alternative antiviral treatments are strongly supported by literature data. Some observational studies and two meta-analyses have suggested the efficacy of HIG at a dosage of 200 IU/kg biweekly in mitigating the risk of congenital CMV infection after primary maternal infection, especially when administered as early as possible after infection during the first trimester [12, 13, 17–19]. However, evidence from previous randomized clinical trials (RCT) is equivocal [12, 13, 20–24], and a recent single-arm Phase III trial including pregnant women with acute CMV infection in their first trimester, to whom HIG was administered at dosage of 200 IU/kg biweekly, could not confirm the benefit of this treatment in preventing maternal–fetal CMV transmission [25]. Therefore, HIG is not routinely recommended for the treatment of women with primary CMV infection in pregnancy [26], as stronger evidence is needed to support its possible applications in this field. In the described case series, HIG was administered as part of a personalized management in the context of unusual clinical scenarios.

In Case 2, the patient received a short course of VCV, interrupted due to intolerance and followed by HIG administration. She ultimately gave birth to an uninfected newborn and did not report any adverse effects related to HIG administration, supporting its potential role in preventing congenital CMV infection, when standard antiviral therapy cannot be continued. This hypothesis would, however, need to be confirmed and explored by future research.

Case 1 and 3 posed an additional challenge, as there is no evidence supporting the best management of immunocompromised pregnant patients with CMV infection. Maternal immunity plays an important role in preventing congenital transmission of CMV; more efficient humoral and cellular immune responses have been observed in pregnant women with primary CMV infection who did not transmit the infection to the fetus, compared to those who transmitted it [27]. Therefore, the transplacental transmission risk is likely higher in either previously or acutely CMV-infected patients with impaired humoral or cellular immune responses, although immunological mechanisms possibly influencing the risk of congenital transmission are only partially known. Based on previous reports, it has been hypothesized that a delayed or absent development of the CD4 + T-cell response is associated with 1) a higher risk of CMV reactivation in immunosuppressed subjects, and 2) a higher risk of transplacental transmission in case of primary CMV infection [27, 28, 35]. However, transplacental transmission rates are not known in this population, with only a few reports focusing on this aspect and a scarcity of data involving solid organ transplant recipients and/or patients undergoing immunosuppressive therapies [28–34].

In Case 1, persistent impairment of CMV-specific CD4 + T-cell immunity likely increased the risk of congenital infection. Although the combination of HIG and valacyclovir may have contributed to viral control, thus preventing the newborn’s infection, the safety and efficacy of this therapeutic approach in such clinical scenarios would need to be further assessed by future research results based on higher levels of evidence [28].

In Case 3, adequate specific CD4 + T-cells responses were documented, but impaired humoral responses, due to the previous anti-CD20 biological therapy [36], justified passive immunization with CMV HIG in addition to VCV, which resulted in detectable levels of anti-CMV IgG in serum after passive HIG administration, providing both neutralizing and ADCC activity. This approach resulted in good tolerability and favorable neonatal outcomes. The absence of an adequate serological response documented for this patient, as well as the positive serology observed after HIG administration—interpreted as being determined by passive exogenous immunization rather than endogenous humoral responses—also posed the challenge of adequately diagnosing and monitoring primary CMV infection in immunocompromised women with impaired cellular or humoral responses. A personalized and close monitoring of CMV reactivation or primary infection should be considered in immunocompromised pregnant women, not only through serology but also through CMV-DNA determination in bodily fluids (blood, urine, saliva). Analysis of the CMV-specific T-cell response may also help to diagnose and monitor the infection in case of isolated IgM detection and lack of IgG response. Of note, the impaired humoral response persisted for more than one year after anti-CD20 biological therapy discontinuation in this case; although hypogammaglobulinemia related to anti-CD20 treatments usually recovers about 12 months after immunosuppressive treatment interruption, cases of prolonged hypogammaglobulinemia up to several years have been reported; the factors related to this condition remain unclear [37].

Importantly, the optimal dosing, duration and timing of HIG therapy remain uncertain. Published therapeutic regimens vary widely, with most studies reporting between two and six administrations among those who applied the 200IU/kg biweekly scheme [12, 13, 17, 23, 38]. Data from Case 3, in which a significant drop in IgG and IgG-related serum activities was observed two weeks after HIG administration, support the appropriateness of the biweekly scheme. In our experience, treatment duration was tailored according to individual clinical circumstances and virological response; HIG was administered with the 200 IU/kg biweekly scheme until a negative amniocentesis for Case 2 (three doses total), and for two doses until the end of the second trimester for Case 3 (in which amniocentesis was not performed due to unfavorable timing). In this case, HIG discontinuation was supported by the persistently negative viremia (despite some fluctuant positive viruria) and considering the low risk of symptomatic congenital infection in case of transmission during the third trimester. The likely higher risk of HIG-related adverse events in the third trimester, as reported in the literature, also supported that decision [39]. Conversely, HIG was administered until the end of pregnancy in Case 1, due to good tolerance and the important challenges associated with infection during the pre- and peri-conceptional period, and the documented inefficient T-cell response.

Finally, concerns have been raised regarding a possible rebound of CMV replication after VCV discontinuation, potentially related to insufficient immune priming during antiviral suppression [40, 41]. Based on these considerations, it has been hypothesized that HIG administration could allow for avoiding the described rebound effect after treatment interruption, by providing passive immunity without inhibiting antigen presentation. On the other hand, it cannot be excluded that exogenous IgG could also reduce antigen exposure through neutralization or epitope masking and potentially modulate endogenous immune priming, thus reducing immune efficiency once the treatment is suspended. Based on these principles, an attempt to stimulate a natural immune response through temporary discontinuation of antiviral therapy before pregnancy was made for Case 1 but led to only a transient recovery of the CD4 + T-cells response and was indeed followed by a rebound in viral load in both saliva and peripheral blood. Given the concomitant positivity of the pregnancy test at this point, HIG and VCV were promptly started again due to the high risk of severe sequelae in case of transmission in the peri-conceptional period. This was followed by a persistently negative viremia until delivery. However, it is not possible to determine whether VCV alone, the addition of HIG to VCV, or their previous suspension to stimulate natural immune responses had the more determinant role in controlling the infection and preventing mother-to-child transmission.

Finally, as in all the described cases patients received valacyclovir during at least one phase of pregnancy, it is not possible to state whether HIG could have independently contributed to the reported positive outcomes; this represents the major limitation of this case series.

## Conclusion

Although HIG is not currently recommended as a first-line strategy for the prevention of congenital CMV infection, as the evidence supporting its effectiveness is limited, this therapeutic option could play a role in selected clinical scenarios.

HIG may represent a therapeutic alternative in cases in which VCV is not tolerated due to its rare but possible adverse effects. Moreover, HIG could be employed to mitigate the risk of congenital CMV as an adjunctive strategy to VCV in case of pregnant immunocompromised women with impaired endogenous responses. The efficacy, most appropriate administration scheme, and safety of HIG employed in these cases should be further explored and analyzed in future prospective studies.

## Data Availability

No datasets were generated or analysed during the current study.

## References

[1] Leruez-Ville M et al (2017) Risk factors for congenital cytomegalovirus infection following primary and nonprimary maternal infection: a prospective neonatal screening study using polymerase chain reaction in saliva. Clin Infect Dis 65(3):398–404. 10.1093/cid/cix337 28419213 10.1093/cid/cix337

[2] Cannon MJ, Schmid DS, Hyde TB (2010) Review of cytomegalovirus seroprevalence and demographic characteristics associated with infection. Rev Med Virol 20(4):202–213. 10.1002/rmv.655 20564615 10.1002/rmv.655

[3] Kenneson A, Cannon MJ (2007) Review and meta-analysis of the epidemiology of congenital cytomegalovirus (CMV) infection. Rev Med Virol 17(4):253–276. 10.1002/rmv.535 17579921 10.1002/rmv.535

[4] P. Palasanthiran, M. Starr, C. Jones, and M. Giles, “Management of Perinatal Infections,” 2014, Accessed: Jun. 22, 2025. [Online]. Available: https://research.monash.edu/en/publications/management-of-perinatal-infections

[5] Chatzakis C, Ville Y, Makrydimas G, Dinas K, Zavlanos A, Sotiriadis A (2020) Timing of primary maternal cytomegalovirus infection and rates of vertical transmission and fetal consequences. Am J Obstet Gynecol 223(6):870-883.e11. 10.1016/j.ajog.2020.05.038 32460972 10.1016/j.ajog.2020.05.038

[6] Maltezou P-G et al (2020) Maternal type of CMV infection and sequelae in infants with congenital CMV: Systematic review and meta-analysis. J Clin Virol 129:104518. 10.1016/j.jcv.2020.104518 32622333 10.1016/j.jcv.2020.104518

[7] Shahar-Nissan K et al (2020) Valaciclovir to prevent vertical transmission of cytomegalovirus after maternal primary infection during pregnancy: a randomised, double-blind, placebo-controlled trial. Lancet 396(10253):779–785. 10.1016/S0140-6736(20)31868-7 32919517 10.1016/S0140-6736(20)31868-7

[8] Egloff C et al (2022) New data on efficacy of valaciclovir in secondary prevention of maternal–fetal transmission of CMV. Ultrasound Obstet Gynecol. 10.1002/uog.26039 10.1002/uog.26039 35900718

[9] Faure-Bardon V, Fourgeaud J, Stirnemann J, Leruez-Ville M, Ville Y (2021) Secondary prevention of congenital cytomegalovirus infection with valacyclovir following maternal primary infection in early pregnancy. Ultrasound Obstet Gynecol 58(4):576–581. 10.1002/uog.23685 33998084 10.1002/uog.23685

[10] Zammarchi L et al (2023) Treatment with valacyclovir during pregnancy for prevention of congenital cytomegalovirus infection: a real-life multicenter Italian observational study. Am J Obstet Gynecol MFM 5(10):101101. 10.1016/j.ajogmf.2023.101101 37516151 10.1016/j.ajogmf.2023.101101

[11] Fitzpatrick A, Cooper C, Vasilunas N, Ritchie B (2022) Describing the impact of maternal hyperimmune globulin and valacyclovir on the outcomes of cytomegalovirus infection in pregnancy: a systematic review. Clin Infect Dis 75(8):1467–1480. 10.1093/cid/ciac297 35438780 10.1093/cid/ciac297

[12] Kagan KO et al (2021) Outcome of pregnancies with recent primary cytomegalovirus infection in first trimester treated with hyperimmunoglobulin: observational study. Ultrasound Obstet Gynecol 57(4):560–567. 10.1002/uog.23596 33491819 10.1002/uog.23596

[13] Kagan KO et al (2019) Prevention of maternal-fetal transmission of cytomegalovirus after primary maternal infection in the first trimester by biweekly hyperimmunoglobulin administration. Ultrasound Obstet Gynecol 53(3):383–389. 10.1002/uog.19164 29947159 10.1002/uog.19164

[14] Fornara C, Furione M, Arossa A, Gerna G, Lilleri D (2016) Comparative magnitude and kinetics of human cytomegalovirus-specific CD4+ and CD8+ T-cell responses in pregnant women with primary versus remote infection and in transmitting versus non-transmitting mothers: Its utility for dating primary infection in pregnancy. J Med Virol 88(7):1238–1246. 10.1002/jmv.24449 26680747 10.1002/jmv.24449

[15] d’Angelo P et al (2024) Evaluation of the in vitro capacity of anti-human cytomegalovirus antibodies to initiate antibody-dependent cell cytotoxicity. Microorganisms 12(7):1355. 10.3390/microorganisms12071355 39065122 10.3390/microorganisms12071355 PMC11278886

[16] Leruez-Ville M et al (2024) Consensus recommendation for prenatal, neonatal and postnatal management of congenital cytomegalovirus infection from the European congenital infection initiative (ECCI). Lancet Region Health – Europe. 10.1016/j.lanepe.2024.100892 38590940 10.1016/j.lanepe.2024.100892 PMC10999471

[17] Seidel V, Hackelöer M, Rancourt RC, Henrich W, Siedentopf J-P (2020) Fetal and maternal outcome after hyperimmunoglobulin administration for prevention of maternal-fetal transmission of cytomegalovirus during pregnancy: retrospective cohort analysis. Arch Gynecol Obstet 302(6):1353–1359. 10.1007/s00404-020-05728-7 32754858 10.1007/s00404-020-05728-7 PMC7584525

[18] Rawlinson WD, Hamilton ST, van Zuylen WJ (2016) Update on treatment of cytomegalovirus infection in pregnancy and of the newborn with congenital cytomegalovirus. Curr Opin Infect Dis 29(6):615–624. 10.1097/QCO.0000000000000317 27607910 10.1097/QCO.0000000000000317

[19] El-Qushayri AE et al (2021) Hyperimmunoglobulin therapy for the prevention and treatment of congenital cytomegalovirus: a systematic review and meta-analysis. Expert Rev Anti Infect Ther 19(5):661–669. 10.1080/14787210.2021.1846521 33148067 10.1080/14787210.2021.1846521

[20] Revello MG et al (2015) Prevention of primary cytomegalovirus infection in pregnancy. EBioMedicine 2(9):1205–1210. 10.1016/j.ebiom.2015.08.003 26501119 10.1016/j.ebiom.2015.08.003 PMC4588434

[21] Nigro G, Adler SP, Torre RL, Best AM (2005) Passive immunization during pregnancy for congenital cytomegalovirus infection. N Engl J Med 353(13):1350–1362. 10.1056/NEJMoa043337 16192480 10.1056/NEJMoa043337

[22] Nigro G (2017) Hyperimmune globulin in pregnancy for the prevention of congenital cytomegalovirus disease. Expert Rev Anti Infect Ther 15(11):977–986. 10.1080/14787210.2017.1398081 29072089 10.1080/14787210.2017.1398081

[23] Devlieger R et al (2021) Serial monitoring and hyperimmunoglobulin versus standard of care to prevent congenital cytomegalovirus infection: a phase iii randomized trial. Fetal Diagn Ther 48(8):611–623. 10.1159/000518508 34569538 10.1159/000518508 PMC8619771

[24] Blázquez-Gamero D et al (2019) Prevention and treatment of fetal cytomegalovirus infection with cytomegalovirus hyperimmune globulin: a multicenter study in Madrid. J Matern Fetal Neonatal Med 32(4):617–625. 10.1080/14767058.2017.1387890 28978246 10.1080/14767058.2017.1387890

[25] Kagan KO, Enders M, Aigner S, Schütze J, Lentze S, Staiger C (2025) Phase 3 clinical trial on the prevention of maternal-fetal cytomegalovirus transmission with specific hyperimmunoglobulin could not demonstrate efficacy. Am J Obstet Gynecol 233(6):e255–e256. 10.1016/j.ajog.2025.07.050 40759386 10.1016/j.ajog.2025.07.050

[26] Khalil A, Heath PT, Jones CE, Soe A, Ville YG, Royal College of Obstetricians and Gynaecologists (2025) Congenital cytomegalovirus infection: update on screening diagnosis and treatment: scientific impact Paper No. 56. BJOG 132(2):e42–e52 39434207 10.1111/1471-0528.17966

[27] Lilleri D, Gerna G (2017) Maternal immune correlates of protection from human cytomegalovirus transmission to the fetus after primary infection in pregnancy. Rev Med Virol. 10.1002/rmv.1921 28008685 10.1002/rmv.1921

[28] Cavoretto P et al (2020) Prenatal management of congenital human cytomegalovirus infection in seropositive pregnant patients treated with azathioprine. Diagnostics (Basel, Switzerland). 10.3390/diagnostics10080542 32751758 10.3390/diagnostics10080542 PMC7459678

[29] Thompson G et al (2018) Analysis of the QuantiFERON-CMV assay, CMV viraemia and antiviral treatment following solid organ transplantation in Western Australia. Pathology 50(5):554–561. 10.1016/j.pathol.2018.04.002 29945729 10.1016/j.pathol.2018.04.002

[30] Hayes K, Symington G, Mackay IR (1979) Maternal immunosuppression and cytomegalovirus infection of the fetus. Aust N Z J Med 9(4):430–433. 10.1111/j.1445-5994.1979.tb04174.x 228648 10.1111/j.1445-5994.1979.tb04174.x

[31] Jones MM, Lidsky MD, Brewer EJ, Yow MD, Williamson WD (1986) Congenital cytomegalovirus infection and maternal systemic lupus erythematosus: a case report. Arthritis Rheum 29(11):1402–1404. 10.1002/art.1780291114 3022760 10.1002/art.1780291114

[32] Armenti VT, Moritz MJ, Davison JM (1998) Medical management of the pregnant transplant recipient. Adv Ren Replace Ther 5(1):14–23. 10.1016/s1073-4449(98)70010-x 9477211 10.1016/s1073-4449(98)70010-x

[33] Von Au M, Schnitzler P, Pöschl J, Koch L, Kräusslich H-G, Böhler T (2012) Delayed detection of cytomegalovirus-specific T-helper cells in a preterm infant following intrauterine exposure to tacrolimus. Clin Lab 58(7–8):811–815 22997983

[34] Evans TJ, Mccollum JPK, Valdimarsson H (1975) Congenital cytomegalovirus infection after maternal renal transplantation. Lancet 305(7921):1359–1360. 10.1016/S0140-6736(75)92264-3 10.1016/s0140-6736(75)92264-3 48947

[35] Marais B, John V, Du Toit M, Mbambo J, John J (2023) Cytomegalovirus haemorrhagic cystitis in a pregnant patient with AIDS. Therapeut Adv Urol. 10.1177/17562872231159531 10.1177/17562872231159531 PMC10034270 36969499

[36] Mikulska M et al (2018) ESCMID Study Group for Infections in Compromised Hosts (ESGICH) Consensus Document on the safety of targeted and biological therapies: an infectious diseases perspective (Agents targeting lymphoid cells surface antigens [I]: CD19, CD20 and CD52). Clin Microbiol Infect 24(Suppl 2):S71–S82. 10.1016/j.cmi.2018.02.003 29447988 10.1016/j.cmi.2018.02.003

[37] Luterbacher F, Bernard F, Baleydier F, Ranza E, Jandus P, Blanchard-Rohner G (2021) Case report: persistent hypogammaglobulinemia more than 10 years after rituximab given post-HSCT. Front Immunol 12:773853. 10.3389/fimmu.2021.773853 35003091 10.3389/fimmu.2021.773853 PMC8727997

[38] Schirwani-Hartl N et al (2023) Biweekly versus monthly hyperimmune globulin therapy for primary cytomegalovirus infection in pregnancy. J Clin Med 12(21):6776. 10.3390/jcm12216776 37959240 10.3390/jcm12216776 PMC10649935

[39] Revello MG et al (2014) A randomized trial of hyperimmune globulin to prevent congenital cytomegalovirus. N Engl J Med 370(14):1316–1326. 10.1056/NEJMoa1310214 24693891 10.1056/NEJMoa1310214

[40] De Santis M et al (2019) Prenatal valacyclovir treatment of fetal citomegalovirus infection: a case series. J Infect 79(5):462–470. 10.1016/j.jinf.2019.08.015 31465779 10.1016/j.jinf.2019.08.015

[41] De Santis M et al (2025) Immune modulation related to high-dose valacyclovir administration for primary cytomegalovirus infection in pregnancy: an insight into virus behavior and maternal serology. Viruses 17(2):157. 10.3390/v17020157 40006912 10.3390/v17020157 PMC11861677

